# Updating the relationship of the Ne/ERN to task-related behavior: A brief review and suggestions for future research

**DOI:** 10.3389/fnhum.2023.1150244

**Published:** 2023-04-04

**Authors:** Sara B. LoTemplio, Clara Louise Lopes, Amy S. McDonnell, Emily E. Scott, Brennan R. Payne, David L. Strayer

**Affiliations:** ^1^Human Dimensions of Natural Resources, Colorado State University, Fort Collins, CO, United States; ^2^Department of Psychology, University of Utah, Salt Lake City, UT, United States; ^3^Department of Psychology, Vermont State University, Johnson, VT, United States; ^4^Interdepartmental Neuroscience Program, University of Utah, Salt Lake City, UT, United States

**Keywords:** error-related negativity, anterior cingulate cortex, cognitive control, post-error slowing, post-error accuracy, performance monitoring, error monitoring, behavior

## Abstract

The error negativity/error-related negativity (Ne/ERN) is one of the most well-studied event-related potential (ERP) components in the electroencephalography (EEG) literature. Peaking about 50 ms after the commission of an error, the Ne/ERN is a negative deflection in the ERP waveform that is thought to reflect error processing in the brain. While its relationships to trait constructs such as anxiety are well-documented, there is still little known about how the Ne/ERN may subsequently influence task-related behavior. In other words, does the occurrence of the Ne/ERN trigger any sort of error corrective process, or any other behavioral adaptation to avoid errors? Several theories have emerged to explain how the Ne/ERN may implement or affect behavior on a task, but evidence supporting each has been mixed. In the following manuscript, we review these theories, and then systematically discuss the reasons that there may be discrepancies in the literature. We review both the inherent biological factors of the neural regions that underlie error-processing in the brain, and some of the researcher-induced factors in analytic and experimental choices that may be exacerbating these discrepancies. We end with a table of recommendations for future researchers who aim to understand the relationship between the Ne/ERN and behavior.

## 1. Introduction

The error negativity, also termed the error-related negativity (Ne/ERN) is a negative deflection in the event-related potential (ERP) waveform that peaks approximately 50 ms after a behavioral error response. Since its discovery over 30 years ago (Falkenstein et al., 1990; Gehring et al., 1993), the Ne/ERN has become widely studied, capturing the attention of cognitive and clinical neuroscientists alike. Today, a google scholar search of the term “error-related negativity” yields nearly 12,000 articles. Its robustness across different contexts and tasks created excitement about the existence of a domain-general error-processing system in the brain, spawning an error-processing literature that was adopted into the growing cognitive control literature at the time. Research has since also demonstrated the Ne/ERN’s clinical utility as a potential biomarker for anxiety disorders (e.g., Olvet and Hajcak, 2008; Meyer, 2017).

While the clinical and motivational factors that influence the Ne/ERN are well-documented (e.g., Hajcak and Foti, 2008; Xiao et al., 2011; Weinberg et al., 2012b,^2015^), the exact nature of how the Ne/ERN might influence behavior remains an interesting unknown (e.g., Gehring et al., 2018). Several theories have emerged to explain how the Ne/ERN may implement or affect behavior on a task (output conditions), but evidence supporting each have been conflicting (Weinberg et al., 2012a; Gehring et al., 2018). We suggest here that by better mapping how the Ne/ERN relates to behavior, we will gain a more enriched understanding of the Ne/ERN itself. Arguably, the brain evolved to guide life-preserving behaviors. Therefore, as some have argued (e.g., Krakauer et al., 2017), in order to truly understand how the brain operates, we must refine our understanding of the brain’s behavioral goals in the first place. While we can learn much about the mechanistic properties and computations of the brain from electroencephalography (EEG) alone (e.g., studies in the sensory prediction literature; Todd et al., 2013; Stefanics et al., 2015), we argue that whenever possible, we should also aim to understand brain activity within the context of associated behavior. Without understanding behavior, we may merely be studying “ERPology” (e.g., Luck, 2014), rather than processes of the mind and brain. Consequently, it will be necessary to further examine and understand how the Ne/ERN and error-processing relate to behavior.

In the following, we review the Ne/ERN literature with a particular emphasis on understanding the Ne/ERN’s relationship to behavior. We will organize our review into two sections, termed “input” and “output.” The former will include an overview of current theories regarding what conditions must be met for an Ne/ERN to occur in the first place (i.e., “input” conditions). In other words- how does a brain recognize an “error?” The latter will outline theories regarding the functional utility of the Ne/ERN as it relates to downstream processes (i.e., “output” conditions). The aim of this section is to ask not what causes the Ne/ERN to occur, but rather why it occurs. In other words—what, if any, behaviors, and processes are initiated in response to the Ne/ERN signal? Are these adaptive? In organizing the review this way, we hope to more clearly delineate what portion of the error-processing stream each emergent theory relates to, hopefully allowing us to resolve seemingly discrepant findings.

We then present several reasons why discrepancies in relating the Ne/ERN to behavior may have occurred in the literature in the first place. First, we argue that some of these issues may be reconciled when we consider the Ne/ERN as part of a multiply determined neural circuit underlying error processing. The Ne/ERN, and error-processing in general, reliably involve multiple areas and processes in the brain. For example, the Ne/ERN has been consistently source-localized to the area of the brain that is likely an integrative informational hub—the anterior cingulate cortex (ACC; e.g., Dehaene et al., 1994; Van Veen and Carter, 2002; Ladouceur et al., 2007). Additionally, advancements in fMRI have documented multiple systems that appear to be critically involved in the implementation of error-processing beyond the ACC (Dosenbach et al., 2008). Furthermore, some EEG oscillatory research on error-processing categorizes the Ne/ERN as part of a larger cognitive control mid-frontal theta substrate, which is also functionally linked to distal areas of the brain beyond the ACC such as the lateral prefrontal cortex, the extrastriate visual cortex, and motor areas (Cavanagh and Frank, 2014). These lines of research strongly suggest that there are intermediate processes between the Ne/ERN and behavior that could potentially explain the discrepancies within the literature.

Furthermore, we discuss the many researcher-driven variations in reporting, task selection, and analysis choices in the Ne/ERN research that could influence interpretations of its relationship to behavior. For example, there is significant variation in Ne/ERN amplitude that is explained by task choice alone (Weinberg et al., 2015). Additionally, there is a strong likelihood that many Ne/ERN studies do not have sufficient internal consistency of Ne/ERN measurements (Clayson, 2020; Clayson et al., 2020). There are also ongoing debates over the best way to even measure and report post-error behavior (e.g., Schroder et al., 2020). Importantly, these researcher-driven discrepancies may exacerbate existing challenges to linking the Ne/ERN to behavior that are driven by the biological considerations discussed above. Therefore, we review these experimental factors and discuss recommendations for future research.

We note that we are not the first to suggest that the Ne/ERN is heterogeneous, multiply implicated, or involved in downstream processes beyond error detection itself (e.g., Cohen, 2014a; Weinberg et al., 2015, 2016; Clayson et al., 2021a). Our primary goal is instead to build on this prior literature by systematically reviewing existing Ne/ERN literature with an eye toward specifically understanding post-error behavior. We will end by making recommendations for future research.

## 2. Input conditions for the Ne/ERN

Perhaps one of the primary considerations of the early Ne/ERN researchers was what the Ne/ERN actually was. In other words, what input, or antecedent, conditions within the brain are necessary or sufficient for the Ne/ERN to occur? It was apparent that the brain was recognizing erroneous responses, but it was not clear how. Several computational accounts emerged, but perhaps the two most prominent theories, that are still under debate today, are the error-detection and conflict monitoring theories. We also will briefly discuss reinforcement learning theory and its relationship to both the Ne/ERN and the related feedback-related negativity (FRN). While the present review focuses primarily on output conditions of the Ne/ERN, it is important to briefly discuss the antecedent conditions that cause it in the first place, as the behavioral outcomes may be theoretically linked to the input conditions that create it.

### 2.1. Error detection

Perhaps one of the earliest hypotheses of the Ne/ERN was that it existed simply as the brain’s error detection network. The computational idea was that the brain, storing the response that should have been made, compared it to the response that was actually made. When it detected an error, it produced the Ne/ERN. Several researchers adopted this early computational account (e.g., Gehring et al., 1993; Coles et al., 2001). However, this theory begets important and puzzling questions- how could a brain that was capable of immediately detecting an error make the error in the first place? Similarly, where exactly was this “correct” response being stored in the brain, and how? Many argued that this account of the Ne/ERN was difficult to reconcile within existing frameworks of human cognition, and that simply ascribing an all-knowing “homunculus” that stored correct responses was not enough (see Gehring et al., 2012).

Later research demonstrating that the Ne/ERN occurred even when participants were not conscious of their errors (Nieuwenhuis et al., 2001; Endrass et al., 2005, 2007; O’Connell et al., 2007), also posed questions for an error-detection framework. What exactly is the brain doing with the error-information if we do not recognize the error? How would humans correct for these errors when there is evidence that conscious recognition of an error is not necessary for the Ne/ERN to occur? However, it is also worth noting that some dispute this claim, and document evidence of conscious awareness indexing Ne/ERN amplitude (e.g., Scheffers and Coles, 2000; Ullsperger and von Cramon, 2006a).

In summary, while this hypothesis gained traction early on, there were enough unresolved questions that many began to doubt its plausibility. Additionally, this hypothesis is difficult to test. Given the technological advancements of the time, how could one know if the brain was “storing” the correct response (though see Charles et al., 2014 for recent developments)? These issues gave rise to another theory of the generation of the Ne/ERN- the conflict monitoring account.

### 2.2. Conflict monitoring

Another early account of the Ne/ERN was the hypothesis that it reflected an innate conflict monitoring process within the brain (e.g., Carter et al., 1998; Botvinick et al., 2001; Yeung et al., 2004). Rather, than detecting errors, these researchers suggested that it was more likely that the Ne/ERN was a manifestation of competing and conflicting responses. In other words, they argued that the Ne/ERN was actually not error-related, *per se*, but rather a measure of how much conflict existed between the desired response and the pre-potent response. Computational modeling suggested that the time course for conflict monitoring in the brain appeared to map on to the time course of the occurrence of the Ne/ERN (Yeung et al., 2004). Yet, strong debate and conflicting results ensued in the years following. While some studies suggest that conflict monitoring may play a role in the generation of the Ne/ERN (e.g., Hughes and Yeung, 2011; Larson et al., 2012; Di Gregorio et al., 2016; for a review see Larson et al., 2014), others suggest complications for this hypothesis (Burle et al., 2008; Masaki et al., 2012).

Perhaps one of the strongest selling points of this research was the existence of what was then termed the correct related negativity (CRN; Vidal et al., 2000, 2003). Early researchers noticed that on some correct trials there still appeared to be an Ne/ERN, just diminished in size. They termed this component the CRN. Importantly, many researchers noticed that the CRN appeared to be prominent on trials of high conflict, such as incongruent trials in the flanker task, especially when the high conflict trials were rare (Bartholow et al., 2005). However, some researchers have suggested that the CRN is not a weaker Ne/ERN, but rather a separate process altogether. For example, Endrass et al. (2012) demonstrated that task difficulty diminished Ne/ERN amplitude while increasing CRN amplitude; this result was also replicated in a later study (Kaczkurkin, 2013). These authors suggest that the CRN may indeed index response conflict as initially thought—but the Ne/ERN is agnostic to conflict and responds only to error detection.

Yet, while many report the existence of the CRN on incongruent trials, many others have failed to find evidence for a CRN at all (e.g., Danielmeier et al., 2009; Gehring et al., 2018). Furthermore, some have found evidence that the CRN may not necessarily be a measure of a response conflict computation itself, but rather an Ne/ERN that occurs at the initiation of an incorrect response, but is quickly corrected (termed a “partial error” e.g., Masaki et al., 2012; Kieffaber et al., 2016). Taken together, the CRN becomes a somewhat weaker argument for conflict-monitoring theory altogether—an Ne/ERN that occurs simply due to the initiation of incorrect motor activity does not necessarily discount the error-detection hypothesis. Though it is worth noting that these findings do not rule out conflict monitoring theory either—it is still possible that an incorrect motor movement could also be a manifestation of response conflict. Further direct experimentation on how response conflict separately affects the Ne/ERN and CRN is needed.

In summary, to date, two of the predominate computational accounts for how the Ne/ERN is generated remain the error-detection and conflict monitoring accounts. Which is more accurate? As the findings from Endrass et al. (2012) suggest, both could be true. Supporting this further, a recent study used electromyography (EMG) to measure partial errors, where participants initiated an incorrect movement, but corrected it before pressing the button. They found that both partial errors and full errors, which are each more likely occur on higher conflict trials, are associated with an increase in midline frontal theta power compared to correct trials. However, they also found a unique pattern of delta activation for full errors alone, dissociating them from conflict detection alone (Cohen and van Gaal, 2014). Gehring et al. (2012) noted over 10 years ago that the debate was far from over as to whether the Ne/ERN’s generation was more closely related to error detection or conflict monitoring, or some other process. If we have learned anything in the intervening years, it is that the answer may not ever come down to a “one or the other” account. Rather, both may be occurring either simultaneously or under different antecedent conditions.

### 2.3. Reinforcement learning theory

Finally, there is one other historical account of what causes the Ne/ERN to occur worth discussing. Reinforcement learning theory (Holroyd and Coles, 2002) suggests that the Ne/ERN is the result of a negative reinforcement learning signal being sent to the ACC from the mesencephalic dopamine system when outcomes are worse than expected. This signal is then utilized by the ACC to improve task performance and avoid future errors. However, evidence linking dopamine and dopaminergic systems to the Ne/ERN has been mixed (for a review see Ullsperger et al., 2014a) with some studies supporting a connection (Falkenstein et al., 2001; Ullsperger et al., 2002; De Bruijn et al., 2004; Stemmer et al., 2007; Seer et al., 2017), others failing to find a clear and straightforward link between the two (Zirnheld et al., 2004; Willemssen et al., 2008; Larson et al., 2015), and still others arguing that there are perhaps only connections to certain dopamine receptors and the ERN (Moustafa et al., 2016; Volpato et al., 2016).

Furthermore, much of the existing work that tested reinforcement learning theory focused on what was then termed the FRN. This component is a negative deflection in the waveform, similar in morphology to the Ne/ERN, that is elicited when participants are given feedback that they made a mistake (Miltner et al., 1997). Researchers initially believed that both the Ne/ERN and the FRN reflected a singular, generalized, error-monitoring system (e.g., Miltner et al., 1997; Holroyd and Coles, 2002; Holroyd et al., 2003). However, over time, accumulating evidence suggested that the feedback-locked component was not a feedback-related negativity, but rather a reward-related positivity that is differentially sensitive to positive reward feedback compared to negative or neutral feedback (RewP; Holroyd et al., 2008; for review see Proudfit, 2015). Therefore, it is unclear how much of the previous work examining the feedback-locked RewP actually pertains specifically to the Ne/ERN.

However, even so, reinforcement learning theory does suggest a certain degree of behavioral adaptation in response to errors, albeit on a different time scale and *via* a different mechanism than simply a behavior change on the next trial. Rather, reinforcement learning theory suggests that, as individuals receive feedback on their performance in real-time, their error-monitoring process transfers from external feedback to internal monitoring, reflecting the learning process. Indeed, previous studies have demonstrated that the Ne/ERN increases as individuals learn the task (e.g., Bultena et al., 2017). Similarly, other studies have shown that when participants are relying more on external feedback to responses, their Ne/ERNs are smaller (Holroyd and Coles, 2002; Nieuwenhuis et al., 2002). However, this still does not explain how properties of the Ne/ERN such as amplitude affect any aspect of subsequent performance, especially in the absence of explicit feedback. Nor does it explain if or how the Ne/ERN implements behavior if the task instructions are clear and little learning is needed.

## 3. Output from the Ne/ERN: Post-error slowing and post-error accuracy

While the input conditions that may generate the Ne/ERN are still under examination, other researchers have attempted to account for what the brain does with the Ne/ERN signal once it occurs—the output conditions. Some of the earliest Ne/ERN researchers suggested that the Ne/ERN served as the brain’s mechanism to correct for such erroneous behavior (e.g., Gehring et al., 1993). Indeed, this would be a convenient narrative to follow; the brain, in some way or another recognizes its mistake, and signals out to correct for this mistake, and then implements strategic adjustment of behavior. Behavioral evidence has long suggested that immediately following errors, humans tend to slow their responses, a phenomenon called post-error slowing (PES; Rabbitt, 1966). Most suggested that PES emerges as a strategy to reduce errors (i.e., Botnivick et al., 2001, for review see Wessel, 2018). Behavioral research had also traditionally shown that participants tend to correct their mistakes immediately following an error (Rabbitt, 1966; Pisella et al., 2000), even when they are instructed not to (Fiehler et al., 2005; Ullsperger and von Cramon, 2006a), suggesting perhaps an automatic and difficult to overcome response in the brain. Perhaps the amplitude of the Ne/ERN would account for such behavioral changes? Unfortunately, the answer is not so simple.

Evidence supporting relationships between the Ne/ERN and behavior adjustment has also been contested over the years (Gehring et al., 2012, 2018; Schroder and Infantolino, 2013). To date, the literature has not converged on one emergent theory that can fully explain behavioral outcomes as they relate to the Ne/ERN. Here, we review the research on post-error behavior and the Ne/ERN in the following section. Here, we first provide an overview of the Ne/ERN’s relationship to overall response accuracy within the experimental task. Next, we relate the Ne/ERN’s relationship to PES, and discuss limitations of this variable as a metric for post-error adjustment. Finally, we cover the less researched outcome of post-error accuracy (PEA) and post-error reduction of interference (PERI).

### 3.1. Ne/ERN and overall accuracy

One of the first questions that Ne/ERN researchers attempted to address was whether any aspect of an individual’s Ne/ERN predicted task accuracy. Some studies have found evidence for larger Ne/ERN amplitudes predicting better overall performance in terms of accuracy (e.g., Gehring et al., 1993; Falkenstein et al., 1995; Maier et al., 2011), but others have not (e.g., Fiehler et al., 2005; e.g., Weinberg et al., 2010; for a review see Gehring et al., 2012). Others theorized that perhaps the Ne/ERN latency, rather than amplitude, might better predict accuracy, such that the sooner the Ne/ERN occurs, the more likely it would be corrected. Again, while some have found compelling evidence for this (e.g., Falkenstein et al., 1991; Fiehler et al., 2005; Hoffmann and Falkenstein, 2010), others have not (Rodrıìguez-Fornells et al., 2002). Given this conflicting evidence, it is possible that the Ne/ERN’s effect on performance would be more immediate, and that it would affect performance on the next trial (i.e., post-error adjustments in behavior), rather than boost overall task accuracy.

### 3.2. Ne/ERN and PES/PEA

One of the most-studied post-error behavior adjustments is PES. A longstanding assumption was that PES occurred as a strategic adjustment to improve behavior. Theoretically, then, a larger Ne/ERN amplitude might predict greater PES. However, there is conflicting evidence as to whether the Ne/ERN is related to this metric either. Some find that larger Ne/ERN amplitudes predicts greater PES (e.g., Debener et al., 2005; Ladouceur et al., 2007; Fischer et al., 2016; Kalfaoğlu et al., 2018; Fu et al., 2019; Steinhauser and Andersen, 2019) while others fail to find any relationship (Gehring and Fencsik, 2001; Hajcak et al., 2003b; Dudschig and Jentzsch, 2009; Riesel et al., 2011; Larson et al., 2016; e.g., Buzzell et al., 2017). One reason why these results might be so confounded, as we will discuss in even greater detail later on, is variation in the level of analysis. Some studies examine how a subject’s average Ne/ERN amplitude affects their average PES (inter-individual analyses), while others examine how Ne/ERN amplitude on a given trial affects reaction time on the next trial within each individual (intra-individual analyses). A recent meta-analysis suggests that there is a small inter-individual effect of Ne/ERN amplitude on the PES, but that this relationship is strengthened when studies leverage an intra-individual approach to examine how Ne/ERN amplitude affects PES on a single-trial basis (Cavanagh and Shackman, 2015). One of the largest Ne/ERN studies to date (*N* = 874) indeed does also find evidence for a relationship between single-trial Ne/ERN amplitude and PES on the subsequent trial (Fischer et al., 2016).

Though these results are promising, there is still considerable debate as to whether PES even relates to behavioral accuracy on the next trial. Many researchers have assumed that PES represents a strategic adjustment to improve accuracy, but this assumption was relatively untested until somewhat recently (Dutilh et al., 2012). While there is some evidence supporting this (e.g., Valadez and Simons, 2018), many have failed to uncover such a relationship (Hajcak and Simons, 2002; e.g., Hajcak et al., 2003a,b, 2004; Hajcak and Simons, 2008; Carp and Compton, 2009). Many researchers now suggest that PES is not an adaptive strategy to improve behavior, but rather a maladaptive disruption caused by the error that actually deteriorates performance, especially at shorter inter-stimulus intervals (ISIs) (Ullsperger and Danielmeier, 2016; Van der Bought et al., 2016; Buzzell et al., 2017), or that PES simply occurs because the error serves as a distraction from the task (e.g., Notebaert et al., 2009; Núòez Castellar et al., 2010). Attempting to reconcile adaptive and maladaptive accounts of PES, Wessel puts forth the adaptive orienting theory (Wessel, 2018), which suggests that PES represents an immediate, maladaptive disruption in task performance at short ISIs. However, when ISIs are longer, downstream processes can continue to completion, and subsequently lead to performance improvements, as manifested by improvements in PEA. Indeed, recent evidence has suggested that PEA increases with increasing inter-trial intervals (ITIs; Dudschig and Jentzsch, 2009). Therefore, in this case, PES may be linked to increased PEA. For this reason, researchers should consider using longer ISIs or response-stimulus intervals when examining PES.

However, troublingly, it is worth emphasizing that recent work suggests that none of the common ways in which researchers calculate PES appear to be reliable measures (Schroder et al., 2020). These researchers show that every method of calculating PES, including the popular measure of robust PES (calculated as difference of the reaction time of the trial after the error and the trial before the error; Dutilh et al., 2012), ultimately has very weak internal consistency. This revelation puts into question whether PES is a useful metric for post-error behavior at all, regardless of whether it is an “adaptive” behavior. Therefore, error-correction, or PEA, might represent a better metric. In most cases, unless only speed is emphasized in task instructions, increased accuracy in a behavioral task is adaptive for that situation (Danielmeier and Ullsperger, 2011; Moser and Schroder, 2012; Schroder and Infantolino, 2013; Schroder and Moser, 2014). This is likely true in real-world situations as well, where errors can sometimes have life-threatening consequences. Yet, few studies investigate PEA at either the inter-individual or intra-individual level (see Table 1 for review). Therefore, while PEA may represent the most direct outcome measure of how the Ne/ERN relates to adaptive behavior for most tasks, there is a relative paucity of information on it.

**TABLE 1:** A review of the literature surrounding relationships between error-related theta/Ne/ERN activity and post-error adjustment.

References	Error-related theta or Ne/ERN?	*N*	Task(s)	Special considerations	# of error trials included in EEG analyses	PES effect sizes between subjects	PEA effect sizes between subjects	PES effect sizes single trial	PEA effect sizes single trial
Cavanagh et al., 2009	Error-related theta	14	Flanker task	Chose two different response-locked analytic windows (−100, 300 ms; 0–200 ms), found that latency of error-related theta also predicted PES, as well as certain inter-channel phase coherences.	*M* = 60.1, SD = 52.2	Not reported	Not reported	−100 to 300 ms: *t*(13) = 2.54, *p* < 0.05*, *d* = 0.68; 0 to 200 ms: *t*(13) = 2.04, *p* < 0.06, *d* = 0.54, n.s.	Not reported
Cohen and Van Gaal, 2013	Error-related theta	19	Visual discrimination task	[N_Ec_/(N_Ee_ + N_Ec_)] to quantify adjustment where *N* = trial number, Ee = error trials followed by error trials, and Ec = error trials followed by correct trials	*M* = 25.5% errors, *SEM* = 2.8%, however, does not specify if this figure is post artifact rejection	*r* = 0.056, *p* = 0.819, n.s.	*r* = 0.68, *p* = 0.002**	Not reported	Not reported
Cohen and van Gaal, 2014	Error-related time frequency signatures (examined all bands- see paper)	64 included in analyses	(1) Color discrimination Simon task (2) Color motion Simon task (3) Color location Simon task (4) Auditory/visual Simon task	Considered partial errors too (see paper), data from all four tasks were pooled together.	*M* = 18.5% errors, SEM = 1.35% included in EEG analyses	Not reported	Not reported	Used correlation coefficients with false discovery correction for all frequencies- found significant cluster for full errors in delta and theta bands. See **Figure 3** in Cohen and van Gaal, 2014.	Not reported
Beatty et al., 2020	Error-related theta	21 included	Color Simon task	Direct comparison of error-related theta and Ne/ERN. We specifically report the theta predicting PES and PEA models (Ne/ERN information is below). Used RSI as a continuous predictor, also used congruency of error trial as interactive predictor term.	Error congruent followed by correct: *M* = 97.00, SD = 40.22 Error incongruent followed by correct: *M* = 158.71, SD = 60.37	Not reported	Not reported	Theta power predicted PES *t*(5197) = 2.509, estimate = 0.035, SE = 0.014, *p* = 0.012, * All other interactions (RSI, congruency) n.s.	Theta power predicted PEA *z* = 4.613, estimate = 0.141, SE = 0.030, *p* < 0.001 *** All other interactions (RSI, Congruency), n.s.
Beatty et al., 2021	Error-related theta	19 included in EEG-behavioral analyses	Color Simon Task	Interested in subthreshold error corrections; also explored lateralized beta power, Also examines Ne/ERN (see below). Includes congruency and next trial congruency as interactive predictive terms.	At least 20 errors to be included. corrected congruent errors (M = 46.526, SD = 33.827); corrected in- congruent errors (M = 85.211, SD = 66.38); uncorrected congruent errors (M = 147.842, SD = 76.547); uncorrected incongruent errors (M = 282.895, SD = 131.858).	Not reported	Not reported	Theta power predicted PES *t*(12,430) = −2.203, estimate = −0.21, SE = 0.009, *p* = 0.028* All other interactions (Congruency, next trial congruency) n.s.	Theta power predicted PEA *z* = 3.291, estimate = 0.070, SE = 0.021, *p* < 0.001*** All other interactions (Congruency, next trial congruency) n.s.
Valadez and Simons, 2018	Error-related theta	10 included in error-related theta and behavior analyses	Flanker task	Reported Ne/ERN relationships as well (listed below)	at least 6 double error and 6 single error per participant	Not reported	Not reported	n.s., effect sizes not reported	*p* < 0.01**, *t* = 15.92
Pfabigan et al., 2021	Error-related theta	35	Flanker task	Study examined effects of buprenorphine on the Ne/ERN/error-related theta. In supplementary materials, reported correlation of deltaPES and FMΘ in error trials specifically. Results also reported below for Ne/ERN.	On average, 18 error trials included.	*r* = 0.51, *p* = < 0.01**	*r* = −0.12, *p* = 0.54	Not reported	Not reported
Cavanagh and Shackman, 2015	Ne/ERN	642 for between subjects, 69 for single trial	Variation of tasks: meta-analysis	Meta analysis, also examined anxiety	Variable	mean *r* = 0.20, *p* < 0.01**	Not reported	mean *r* = 0.52, *p* < 0.01**	Not reported
Fischer et al., 2016	Ne/ERN	874	Speeded arrow Eriksen flanker task	Also examined gender differences	*M* = 142	n.s., effect size not reported	Not reported	β = -0.33, CI = -0.22– -0.44, *p* < 0.001***	Not reported, but PES did correlate to PEA
Buzzell et al., 2017	Ne/ERN	23 included	Two-choice perceptual decision-making task, 1,680 trials total	Sorted according to RSI as well	*M* = 21.84% errors on behavioral task (SE = 1.58%) Artifact correction rather than rejection (channel interpolation), all errors included	Not reported	Not reported	n.s., *p* > 0.08	n.s. *p* > 0.06
Kalfaoğlu et al., 2018	Ne/ERN	11 included in Ne/ERN analysis, 19 in behavioral	Copy-typing task	Participants completing continuous typing task (no specific “trials”)	Corrected error *M* = 71, Uncorrected error *M* = 36	Not reported	Not reported	β = −0.18, *t*(11) = −2.19, *p* = 0.03*	β = −0.31, *t*(11) = −3.38, *p* < 0.001***
Valadez and Simons, 2018	Ne/ERN	10 included in Ne/ERN- behavior analyses	flanker task	Reported error-related theta relationships as well (listed above)	At least 6 double error and 6 single error per participant	Not reported	Not reported	N.s., effect sizes not reported	N.s., effect sizes not reported
Fu et al., 2019	iNe/ERN (intra-cranial)	29 patients, isolated and included 399 dACC neurons and 431 pre-SMA neurons after artifact rejection	Color-naming Stroop task	Single-unit recording of neurons and intra-cranial EEG in patient populations	At least 7 errors per neuron	Not reported	Not reported	N.s., but did median split of high PES trials and low PES trials to look at differences in iNe/ERN amplitude	Not reported
Beatty et al., 2020	Ne/ERN	*N* = 21 included	Color Simon task	Direct comparison of error-related theta and Ne/ERN. We specifically report the Ne/ERN predicting PES and PEA models (error-related theta information is above) Used RSI as a continuous predictor, also used congruency of error trial as interactive predictor term.	Error congruent followed by correct: *M* = 97.00, SD = 40.22 Error incongruent followed by correct: *M* = 158.71, SD = 60.37	Not reported	Not reported	Ne/ERN predicted PES *t*(5201) = -0.357, estimate = -0.005, SE = 0.014, *p* = 0.721. Interaction between Ne/ERN × Congruency on PES: *t*(5351) = −2.145, estimate = −0.058, SE = 0.27, *p* = 0.03*, such that a larger Ne/ERN leads to slower RT after incongruent, but not congruent errors	z = -1.551, estimate = -0.049, SE = 0.032, *p* = 0.121, n.s.
Kirschner et al., 2021	Ne/ERN and Pe	*N* = 63	Error awareness task	Examined whether error awareness related to post-error behavioral adjustment	Not reported	Not reported	Not reported	N.s.	Not reported
Beatty et al., 2021	Ne/ERN	*N* = 19 included in EEG-behavioral analyses	Color Simon task	Interested in subthreshold error corrections; also explored lateralized beta power. Also examines error-related theta (see above). Includes congruency and next trial congruency as interactive predictive terms.	At least 20 errors to be included. Corrected congruent errors (M = 46.526, SD = 33.827); corrected in-congruent errors (M = 85.211, SD = 66.38); uncorrected congruent errors (M = 147.842, SD = 76.547); uncorrected incongruent errors (M = 282.895, SD = 131.858).	Not reported	Not reported	Ne/ERN predicted post-error slowing *t*(13,250) = −3.866, estimate = 0.35, SE = 0.009, *p* < 0.001*** All other interactions (Congruency, next trial congruency) n.s.	Ne/ERN does not predict PEA, n.s. All other interactions (Congruency, next trial congruency) are also n.s.
Li et al., 2022	Ne/ERN	32	Flanker task	Also used multi-variate pattern analysis to understand post-error behavior, as well as the interaction between congruency and Ne/ERN/behavior relationships.	*M* = 54 congruent errors, 155 incongruent errors	Not reported	Not reported	Main effect of Ne/ERN-PES = n.s., *p* > 0.09, interactive effect of Ne/ERN-PES-Congruency = n.s.	Main effect of Ne/ERN-PEA = n.s., *p* = 0.259, interactive effect of Ne/ERN-PEA-Congruency = n.s.
Pfabigan et al., 2021	Ne/ERN	35	Flanker task	Study examined effects of buprenorphine on the Ne/ERN/error-related theta. In supplementary materials, reported correlation of deltaPES and midline frontal theta in error trials specifically. Results also reported above for error-related theta.	On average, 18 error trials included.	*r* = −0.283, *p* = 0.13	*r* = −0.34 *p* = 0.07	*r* = −0.283, *p* = 0.13	Not reported

Post-error slowing (PES) and post-error accuracy (PEA) are examined. Larger error-related activity is treated as a “positive” effect. Therefore, positive relationships indicate longer response times or increased accuracy. We assume that most studies examining the relationship between the Ne/ERN and PES/PEA published before 2015 were included in a 2015 meta-analysis (Cavanagh and Shackman, 2015), and therefore only report the results of the meta-analysis here. Note that this meta-analysis did not include papers that examined only error-related theta, so papers using this metric before 2015 were still included. Only papers that specifically examine the Ne/ERN or error-related theta and its relationship to PES/PEA are included. Therefore, papers that discussed pre-stimulus, conflict related theta its relationship to behavioral adjustment are not included, nor papers that examine connectivity and its relationship to post-error behavior.

*p < 0.05, **p < 0.01, ***p < 0.001.

n.s., non-significant; #, Number; Pe, Positivity following an error.

### 3.3. Ne/ERN and PERI

It is also worth briefly discussing the less commonly used measure of post-error reduction in interference (PERI; Ridderinkhof, 2002; Danielmeier and Ullsperger, 2011). Previous work suggested that, following an error, the performance differences between congruent and incongruent trials is reduced (e.g., Ridderinkhof, 2002). While it is challenging to relate PERI to a single trial Ne/ERN amplitude, some research has indeed found that average Ne/ERN amplitude can predict the degree of congruency-related discrepancies in performance (e.g., Maier et al., 2011). Yet, further work is necessary to explicitly examine how Ne/ERN amplitude might relate to PERI. Nevertheless, this phenomenon highlights the reality that post-error behavior adjustment may occur in subtler ways than overt behavioral accuracy on the next trial, as we further discuss later in the manuscript. However, note that there have been complications in using this measure, as it originally failed to take into account the congruency of the previous trial (Van der Borght et al., 2014). Similarly, PERI is subject to the same criticisms as PES in that it is unclear whether this adjustment is adaptive or maladaptive.

## 4. Why is the evidence so mixed? The neurobiological factors

As discussed, there is still contradictory information about the Ne/ERN’s behavioral purpose and utility. As a field, we need to reconsider the framework from which we approach the Ne/ERN. Error-processing encompasses many different situations, behaviors, goals, and individuals. As the process of error detection is critical to an organism’s success and survival, it’s likely that the brain, as many complex systems do, has redundancy (Gunderson, 2001; Sporns et al., 2004) built into it to protect such an important process. Therefore, there may be multiple input and output conditions to the Ne/ERN that produce similar, but not identical results. Indeed, the brain and underlying error-processing structures seem biologically suited to coordinate and integrate complex and differing error scenarios. Here we argue that, due to the ubiquity of error-processing in the brain and the high involvement of error-processing networks in many different processes, the Ne/ERN may be multiply involved. Rather than a specific instantiation of behavior, it may instead act as an alarm signal of sorts (Cavanagh and Frank, 2014), that calls for behavior implementation in downstream areas. In this way, it may be naturally difficult to relate to any one specific behavior.

### 4.1. High connectivity of the ACC and multiple contributions to error processing

The Ne/ERN appears to be primarily generated from the dorsal anterior cingulate cortex (ACC; Gemba et al., 1986; Miltner et al., 2003; Hester et al., 2004; O’Connell et al., 2007). The ACC is the anterior portion of the cingulate cortex, a prominent strip of cortex that surrounds the corpus callosum. Previous researchers have divided the cingulate into many different subdivisions based on various qualifications (for a review see Shackman et al., 2011). For clarification’s sake, when we refer to the ACC throughout this document, we specifically refer to what is sometimes termed the anterior mid-cingulate cortex, or mid-cingulate cortex (e.g., Cavanagh and Frank, 2014). This is the area that was previously considered the “cognitive division” of the cingulate and termed the dorsal ACC (Bush et al., 2000), though now some further divide this area into the anterior mid-cingulate and the posterior mid-cingulate cortex (e.g., Dosenbach et al., 2008).

The ACC is involved in a variety of cognitive processes. While it is nearly always implicated in theories of cognitive control (e.g., Nee et al., 2007; Shackman et al., 2011; Metzler-Baddeley et al., 2012; Niendam et al., 2012), what exact functions this includes appears to be vast and varied. For example, evidence has suggested that the ACC is involved in conflict monitoring (Botvinick et al., 2001; Botvinick, 2007), decision-making (Bush et al., 2002; Gehring and Willoughby, 2002; Kennerley et al., 2006; Botvinick, 2007), reward learning (Hadland et al., 2003; Amiez et al., 2005), and error-processing (Carter et al., 1998; Gehring and Fencsik, 2001; Garavan et al., 2002). The ACC also is involved in pain perception (Rainville et al., 1997; Hutchison et al., 1999; Vogt, 2009), empathetic perception of pain in others (Lloyd et al., 2004), social pain (Eisenberger et al., 2003; though see Wager et al., 2016; for a review see Rotge et al., 2015) and negative affect (Bush et al., 2000; Etkin et al., 2010). Interestingly, there is even evidence suggesting that the ACC is connected to the cardiovascular system *via* the vagus nerve (Thayer et al., 2012), potentially integrating information about extant stressors. In addition to its involvement in numerous processes, it is also functionally connected with other cortical and subcortical regions throughout the brain (Margulies et al., 2007; Torta and Cauda, 2011), and a long history of primate research has suggested that it is highly structurally inter-connected as well (e.g., Pandya et al., 1981).

Given its extensive connection to many areas of the brain and body, multiple theories have emerged arguing that the ACC acts as an integrative hub. For example, the adaptive control hypothesis suggests that there is no distinct boundary between cognitive and affective processing in dorsal ACC, but instead that the ACC integrates information regarding both processes in order to allocate top-down control (Shackman et al., 2011). Shenhav et al. (2013) describe the ACC as essentially the “treasurer” of the brain—computing how much cognitive control a given task will “cost,” and the value expected to be received for doing well by incurring that cost. It then uses this information to prioritize the allocation of resources to various cognitive tasks. These authors argue that the ACC is well situated to receive both perceptual and emotional inputs that may help to determine the “state” associated with the task, and its importance.

While modern theories of the ACC as an integrative hub do much to explain its purpose despite the multitude of connections and predictions, the Ne/ERN literature may be lagging in its consideration of these models. As the cingulate likely does act as a complex integration center for a large variety of information, we must be careful not to assume that the integration of all of these varying inputs will always lead to the same output (i.e., an Ne/ERN). Furthermore, aside from the heavy involvement of the ACC in numerous processes, evidence suggests that multiple networks beyond the ACC are involved in the brain’s error-processing and goal-maintenance systems (e.g., Dosenbach et al., 2006, 2007, 2008; Ullsperger et al., 2014a; Neta et al., 2015). These networks are responsible for implementing cognitive control in general, of which error-processing is one piece (Dosenbach et al., 2006, 2007, 2008).

For example, the dual-system model of cognitive control (Dosenbach et al., 2008) suggests that two brain networks could be responsible for the overall process of error monitoring. This model suggests that the cingulo-opercular network (CON) is responsible for the stable, set-maintenance of the task, while the fronto-parietal network (FPN) is involved in the initiation and adjustment of control from trial to trial (for further review see Dosenbach et al., 2006, 2007, 2008). Therefore, while the CON structures may be well suited to maintain the task long term *via* processes such as error-monitoring, the FPN is perhaps well positioned to receive the information and then implement the necessary behavior adjustments on a trial-by-trial basis^1^. By this logic, error-processing and implementation of error-correction may recruit multiple neural resources beyond the ACC.

Consistent with this, research has long suggested the involvement of other brain areas beyond the ACC to the generation of the Ne/ERN (e.g., see Ullsperger et al., 2014a)—specifically the lateral pre-frontal cortex (PFC). For example, patients with lateral PFC lesions or damage have a decreased Ne/ERN compared to control (Gehring and Knight, 2000; Ullsperger et al., 2002; Ullsperger and von Cramon, 2006b), and it appears that both networks, according to the original work by Dosenbach et al. (2007), show increased activity in response to errors. Specifically, error-related activity in the ACC is followed by a boost in dorsolateral prefrontal cortex (DLPFC) activity (Kerns et al., 2004). The DLPFC, an area of the FPN, is known to be related to successful engagement of working memory to perform well on tasks (e.g., Barbey et al., 2013). Similarly, researchers have found that error-related midline frontal theta, which we will discuss in further detail below, predicts connectivity between DLPFC and an area of the CON known as the mid-cingulate cortex (Buzzell et al., 2017). This all together suggest that the CON communicates errors, while the FPN adjusts subsequent behavior accordingly.

Some researchers have even suggested an even broader profile of error-monitoring in the brain (e.g., Neta et al., 2015) of over 40 regions of interest implicated in error-processing on various time-scales. This provides further evidence that error-related activity is broadly distributed in the brain, and suggests differences in tasks and contexts across experiments may differentially affect how we observe the Ne/ERN, as Weinberg et al. (2015) have put forth. In summary, while error responses were initially relegated to just the ACC in many cognitive control models, there is compelling evidence that both the FPN and CON and potentially more structures transmit information about errors, and what to do about them. This suggests again that consideration of how the Ne/ERN affects behavior necessitates understanding how various other systems, beyond the ACC alone, interact when errors occur.

### 4.2. Alternative ways of conceptualizing and quantifying the Ne/ERN: Error-related theta

Alongside more recent developments in EEG methodology and analytic techniques, cognitive control researchers have looked to time-frequency information in the brain to better understand the relationship between error-processing and behavior. Early studies indeed suggested that the Ne/ERN appeared to be part of a theta oscillation (Luu and Tucker, 2001; Makeig et al., 2002; Luu et al., 2004), with studies routinely confirming the presence of both theta phase consistency and increases in theta power during the Ne/ERN’s onset (e.g., Yordanova et al., 2004; Trujillo and Allen, 2007; Cavanagh et al., 2009; Zavala et al., 2016). Research also demonstrated that the ACC also generates theta oscillations (Asada et al., 1999; Tsujimoto et al., 2006). Thus, a biologically plausible account of the Ne/ERN as a substrate of a theta oscillation emerged.

In light of these developments, some have argued that quantifying the Ne/ERN in time-frequency space may lead to better understanding of its relationship to behavior (e.g., Valadez and Simons, 2018), as time-frequency measures retain non-phase locked as well as phase-locked information (Cohen, 2014b; McKewen et al., 2020). Time-frequency analyses have yielded some modest success in coupling error-related theta to behavior (see Table 1). Specifically, there is evidence that both PES within-subjects and PEA between subjects correlates with error-related theta activity. At times, studies have found relationships between error-related theta and post-error behavior while not finding these relationships with the Ne/ERN within the same dataset (e.g., Valadez and Simons, 2018; Pfabigan et al., 2021).

Still, research coupling theta and behavior is limited, and few studies examine both PEA and PES, especially at the intra-individual level. Furthermore, a common theta substrate is present in numerous cognitive processes beyond error-processing (Cavanagh et al., 2012; Cavanagh and Frank, 2014; Ullsperger et al., 2014a,b). While these cognitive control processes are similar in many ways, each may require distinctly different behavioral outcomes. In some cases, these outcomes are directly contradictory to one another. For example, in some situations, the “need for control” may mean slowing down behavior- in others, it means anticipating a switch in task demands (Cooper et al., 2019) and in this case speeding up responses. Thus, the mechanism for how theta might coordinate such discrepant behaviors is unclear. Altogether, preliminary evidence does not suggest a direct relationship between theta and behavior, even in the case of error-processing alone. As some suggest (e.g., Cavanagh and Frank, 2014), downstream processes may need to occur between the theta signal and the eventual behavioral implementation.

Accumulating evidence indeed supports this notion. Several researchers have documented inter-channel coupling between medial frontal areas and lateral frontal areas following errors (e.g., Cavanagh et al., 2009; Van de Vijver et al., 2011; Anguera et al., 2013, for a review see Cavanagh and Frank, 2014). Buzzell et al. (2019) also found EEG evidence that post-response MFC-LFC connectivity was associated with correct responses on the next trial. While we should be cautious about interpreting functional connectivity in EEG research, this research appears to complement ongoing fMRI frameworks of error processing, again suggesting that the ACC may be communicating directly to recruit resources from the DLPFC (e.g., Kerns et al., 2004).

In addition to accounting for the biological plausibility of our models of error processing, this literature suggests that we should account for other midline frontal theta ERP components when we consider the Ne/ERN. Several studies have demonstrated that pre-stimulus theta relates to behavioral accuracy on a trial (e.g., Cohen and Cavanagh, 2011; Buzzell et al., 2019), and models of compensatory error processing suggest that pre-response conflict signals such as the anterior N2 might also inform us about the utility of the Ne/ERN and similar components (e.g., Grützmann et al., 2014). Similarly, researchers should also consider the related mismatch negativity (MMN; Näätänen et al., 1978), which also occurs in the theta band (e.g., Javitt et al., 2018). The MMN typically occurs in the auditory modality when an individual hears a sound that deviates in tone from the previous sequence of sounds (for review see Näätänen et al., 1993), and is morphologically similar to the ERN. Functionally thought to represent a prediction error in the brain (for review see Garrido et al., 2009; Bendixen et al., 2012), its relationship to the ERN is not entirely clear, with examples of the two components covarying (e.g., Ding et al., 2022), and dissociating under various circumstances (e.g., Sasidharan et al., 2019). Therefore, while the two components are currently considered distinct, it may be important to consider where the two boundaries between these components may become blurred, particularly in the auditory domain. Implications for future research are discussed in a later section.

In summary, more recent neurobiological research has demonstrated that error-processing has several underlying neural circuits, and is involved in a number of different cognitive processes. Therefore, it may naturally be difficult to relate any given error response to a singular, definitive behavioral outcome. Fortunately, there are several “experimental factors” that we can leverage to overcome these challenges.

## 5. Why is the evidence so mixed?: The experimental factors

We now review several experimental factors that may exacerbate the discrepancies seen in the literature. We argue that it has been difficult to link the Ne/ERN to any specific post-error behavior due to variation in experimental design, analytic choice, and the tendency to quantify “errors,” in such a way that excludes more naturalistic movements. Fortunately, many of these challenges are solvable. Below, we relate these challenges to the aforementioned “neurobiological” factors and discuss recommendations for future research. We make detailed recommendations at the end of each section, but compile them into Table 2 for easy reference. Similarly, we compile a list of answerable but pressing questions about the Ne/ERN and behavior in Box 1. We believe answers to these questions, *via* the use of both experimental and meta-analytic techniques, would provide some of the most useful insight into the relationship between the Ne/ERN and behavior.

**TABLE 2:** As discussed, in addition to the variability in the Ne/ERN’s relationship to behavior that is likely “naturally” caused by various biological subsystems, there are a variety of researcher decisions that may exacerbate existing discrepancies.

Consideration	Solution(s)
Many researchers do not report the Ne/ERN’s relationship to behavior	For the benefit of the field, we recommend that authors report the Ne/ERN’s relationship to task-related behavior whenever possible. These can easily be added as supplementary materials or exploratory analyses, even if they weren’t pre-registered. Report effect sizes of the Ne/ERN-behavior relationship for use in future meta-analyses
There is considerable debate about the reliability of PES measures as well as their functional importance to post-error behavioral adjustment.	Researchers should report the Ne/ERN’s relationship to PEA in addition to PES If you choose to report PES, consider reporting internal reliability of this measure
Subject-level and single-trial reports of brain-behavior relationships often represent completely separable processes. Yet, many researchers only report subject-level relationships.	When reporting the Ne/ERN’s relationship to behavior, researchers should report single trial (intra-individual) relationships to behavior whenever possible, in addition to subject-level (inter-individual) relationships whenever possible. Furthermore, researchers should ensure to include analyses that report the Ne/ERN’s relationship to PEA—both at the intra-individual and inter-individual levels.
There are many “researcher degrees of freedom” that may influence results	Pre-register hypotheses and methods Follow recommended ERP reporting guidelines outlined in Keil et al. (2014) Try to control for other known influences when possible (i.e., controlling for motivation when examining the Ne/ERN and anxiety)
There is a wide variety in tasks used to elicit the Ne/ERN. The Ne/ERNs elicited from these tasks can have varying reliabilities based on task.	Always report, in as much detail as possible, what task was used to elicit the Ne/ERN. Work together to create standardized data sets for each common Ne/ERN task, as demonstrated by Imburgio et al. (2020) When it makes experimental sense, use these standardized tasks outlined above.
Internal consistency of Ne/ERN scores is not often reported and is likely low in many existing studies.	Report internal consistency of your Ne/ERN scores If experimentally appropriate, one consideration for increasing reliability and statistical power to detect effects is to aim to increase the number of error trials in each condition (and therefore waveform) through experimental design (e.g., Thigpen et al., 2017; Boudewyn et al., 2018).
Errors are often studied within the context of simple two-choice response tasks. However, “real-world” errors are often less binary than this.	We encourage researchers to continue using EMG as a methodology to further understand “partial” errors in a simple two choice response task We also encourage researchers to study the Ne/ERN in more naturalistic movement paradigms when possible. We also encourage consideration of tasks that might elicit an Ne/ERN in the absence of overt movement (e.g., Spinelli et al., 2018)
Some researchers measure error-related theta in the time-frequency domain, while others measure the Ne/ERN in only the time domain.	Researchers should aim to compare error-related theta and the Ne/ERN’s relationships to post-error behavior (e.g., Beatty et al., 2020). To this end, as advances in computational power have made time-frequency analyses increasingly viable, efforts should be made to share scripts, pipelines, and analysis expertise to those interested in conducting or adding time-frequency analyses.

In this table, we summarize these “experimental” considerations and then outline recommended solutions.

**BOX 1 Pressing, answerable questions about the Ne/ERN’s relationship to behavior.**

(1) In the same samples, do inter-individual and intra-individual examinations of relationships between Ne/ERN amplitude and PEA show the same patterns?

(2) Are these relationships better described by error-related theta, rather than the Ne/ERN?

(3) Does task choice affect trial-by-trial relationships between Ne/ERN amplitude and post-error behavior? For example, do certain tasks show the Ne/ERN predicting post-error slowing while other tasks show the Ne/ERN predicting post-error speeding?

(4) Do error types affect trial-by-trial relationships between Ne/ERN amplitude and post-error behavior? For example, is the relationship between the Ne/ERN and post-error behavior different for “fast” errors vs. “slow” errors?

(5) How does the Ne/ERN amplitude in more “naturalistic” errors relate to behavior change or adjustment? For example, to what extent do partial errors as measured by EMG relate to accuracy on the next trial?

(6) Can the Ne/ERN’s relationship to behavior adjustment be better described by drift-diffusion models?

### 5.1. Variations in experimental and Ne/ERN analytical choices

Arguably one of the primary considerations for why it has been difficult to link the Ne/ERN to any sort of behavior adjustment is the lack of standardization in data collection and reporting procedures in the Ne/ERN literature. The advent of the open science movement has demonstrated that researcher “degrees of freedom” can heavily influence results of studies (e.g., Simmons et al., 2011). This applies directly to the Ne/ERN and its relationship to behavior—a recent review examined how various analytic pipelines affected Ne/ERN outcomes (Sandre et al., 2020). They varied references, baselines, amplitudes, and electrode site scorings for a total of 72 different processing pipelines. They found that not only did these choices affect Ne/ERN amplitude, internal consistency, and test-retest reliability, but that it also affected the relationships between Ne/ERN amplitude and post-error behavior. Similarly, another study found that variations in methodological choices impacted the strength of the relationship between anxiety disorders and the Ne/ERN (Klawohn et al., 2020). Therefore, while there still may be significant debate over the “best” way to measure the Ne/ERN that may vary depending on the experimental goals, one thing is strikingly clear: analytic choices matter, and these choices should be made thoughtfully. To this end, we strongly recommend that researchers use open science practices—and share not just their existing datasets, but also their analysis pipelines and their *a priori* hypotheses (e.g., LoTemplio et al., 2020). We also strongly recommend that researchers follow standard protocol outlined in Keil et al. (2014) for reporting analysis pipelines.

While much focus has been devoted to eliminating sources of researcher bias in the analytical process, variations in Ne/ERN results can also occur from seemingly benign choices such as experimental tasks. While recent research has found strong evidence for convergent validity of the Ne/ERN across tasks (Riesel et al., 2013), Weinberg et al. (2015) point out that ranges of correlations from 0.33 to 0.66 still represent a large amount of variance that may be explained by task differences. For example, though there is strong convergent validity across tasks, Riesel et al. (2013) did show that the flanker task yielded the highest split-half reliability score (0.81) compared to other tasks such as the Stroop (0.69) and the Go/NoGo (0.60). Perhaps most importantly, variations among tasks might differentially affect behavior as well as Ne/ERN amplitudes, complicating our understanding of the Ne/ERN and behavior relationship. For example, a recent meta-analysis of obsessive compulsive disorder (OCD) studies revealed no relationship between OCD and Ne/ERN amplitude for non-conflict tasks, but a robust relationship between these variables did exist in conflict tasks (Riesel, 2019). It is therefore possible that the relationships between the Ne/ERN and behavior such as PEA could similarly change depending on the task. Certain tasks may indeed warrant differential behavior adjustment after errors. For example, if a participant is instructed to respond as fast as they can to a single stimulus, a successful error adjustment might require them to speed their response. On the other hand, in a high conflict task with an accuracy emphasis, such as the Stroop task, PES may be a more adaptive behavior. Finally, certain task modalities, such as auditory discrimination tasks, could even tap into other, unintended ERP components that are closely related to the ERN, such as the MMN (Näätänen et al., 1978), as previously discussed.

Additionally, even within the same task, not all errors are created equally. For example, some errors may occur due to one’s inability to override a pre-potent response, while others may occur due to a lapse in sustained attention, as van Driel et al. (2012) suggest. Furthermore, there is evidence suggesting that Ne/ERN amplitude is more sensitive to fast “impulsive” errors rather than slower errors (e.g., Stahl et al., 2020). As we will discuss in more detail below, there may also be differences in errors and “partial errors” in which the participant initiated an incorrect movement, but changed courses and corrected it before making their response (Cohen and van Gaal, 2014). Importantly, these differences in task type and error type likely engage different underlying neural systems (e.g., Cavanagh et al., 2010; Weinberg et al., 2015). So far, these choices have also only covered differences in tasks/task goals or instructions. However, we can also consider how variations in task difficulty, even within the same task, might also complicate relationships to behavior. For example, one study found that both Ne/ERN and CRN amplitude were influenced by task difficulty, and that overall reaction times and error rates were also influenced (Kaczkurkin, 2013). As we discuss in more detail later on, care is needed to understand how these difficulty effects might influence relationships between the Ne/ERN and task-related behavior.

Finally, analytic choices about the errors themselves can influence interpretations. For example, though previous studies have suggested that as few as eight error trials are needed to reliably estimate Ne/ERN amplitudes (e.g., Meyer et al., 2013), recent work has shown that this estimate may not be sufficient in many experimental contexts (Clayson, 2020; Clayson et al., 2020). Instead, in theme with many other aspects of Ne/ERN research reviewed here, the internal consistency of the Ne/ERN can vary dramatically depending on task choice and participant population (i.e., patient vs. healthy controls). Like other ERP components, the internal consistency of Ne/ERN estimate dramatically increases with the number of trials (Clayson, 2020; Clayson et al., 2020), as does the statistical power to detect effects (Boudewyn et al., 2018). We recognize that the inclusion of additional trials presents challenges for Ne/ERN researchers, as we are not in control of how many errors participants make. Given that many participants perform well on simple two-choice tasks that are common in the Ne/ERN literature, even under speed instructions, hundreds of trials are often needed to produce a small proportional amount of errors. Researchers must balance the need for increased error counts with participant fatigue. Therefore, if the number of errors can not be increased, we echo Clayson (2020)’s recommendations to at least report internal consistency.

Given these considerations, the field would benefit from standardization of methods—from task selection, to data collection and processing. There have been recent useful and admirable efforts to create normative data for Ne/ERN differences scores using the arrows flanker task (e.g., Imburgio et al., 2020). These efforts are encouraging, and should be repeated for other common Ne/ERN tasks. This is not to say that researchers should always utilize one task over another, and indeed it is important to use a variety of tasks to understand the generalizability of error-processing. Rather, differences in Ne/ERN morphology according to task choice may inform much of our understanding about error-processing and its relationship to both behavior and pathology—but only if we carefully document, norm, and compare these varying datasets.

### 5.2. Variations in the measurement and reporting of brain-behavior relationships

In a comprehensive review, Weinberg et al. (2012a) highlight the predominance of inter-individual analyses in the Ne/ERN literature as opposed to intra-individual analyses. Most studies, they show, tend to examine whether a person with a larger Ne/ERN shows more PES (inter-individual variability), rather than looking at whether a single-trial Ne/ERN elicits PES on that following trial within a subject (intra-individual variability). Yet, very few studies report both inter and intra-individual relationships between brain and behavior, as we review in Table 1. While inter-individual differences and intra-individual differences often reflect the same underlying process or relationship, occasionally the two completely dissociate (e.g., Simpson, 1951; Kievit et al., 2013; LoTemplio et al., 2021). Considering that we know that the Ne/ERN is indexed quite strongly by individual differences in anxiety and cognitive ability (e.g., Weinberg et al., 2012b; Meyer and Hajcak, 2019), analyses of between subject effects of the Ne/ERN on behavior may tell us more about these individual differences themselves than the actual coupling of the brain activity to behavior. This might even extend to experimental variables-for example, a higher difficulty task might simultaneously affect Ne/ERN amplitude and behavior. Yet, by merely using inter-individual analyses alone, it is impossible to know if a reduction in Ne/ERN amplitude is even related to behavior on the next trial, or if both of these measures are merely separately affected by task difficulty. Furthermore, averaging all single trial Ne/ERN scores together to form a grand average ERP, for comparison to mean reaction time, rests on the assumption that the Ne/ERN remains stable throughout the course of the experiment (Clayson et al., 2021b).

Indeed, some studies have had luck in linking PES to Ne/ERN amplitude at the single-trial level. In a recent meta-analysis of Ne/ERN and behavioral data, Cavanagh and Shackman, (2015; see Table 1 for summary of meta-analysis results) show that intra-individual analyses of Ne/ERN and PES do indeed yield a significant relationship, such that the larger the Ne/ERN amplitude, the greater the PES. Comparably, the inter-individual analyses yielded a much smaller, albeit still present, relationship between the two. Therefore, we recommend that future Ne/ERN studies should report intra-individual effects on behavior as often as possible in addition to the classic inter-individual effects, as data on the former is relatively sparse. We recognize that conducting these intra-individual analyses may not make sense for every Ne/ERN experiment. However, if a researcher is already reporting relationships between the Ne/ERN and behavior at the subject level, it is relatively straightforward to then compute the single-trial relationships, as Matlab retains epoched single trial ERP data in order to compute the grand average waveform. We also recognize that researchers may be concerned about adding another analysis to an already dense study—in this case we might recommend reporting this information in supplementary materials or as an exploratory analysis, or publicly sharing data for other researchers to conduct the analyses.

Finally, as previously discussed, PEA may represent a better metric for post-error adjustment than PES. Yet, very few papers have directly examined relationships between Ne/ERN amplitude and accuracy on the next trial (PEA; see Table 1 for review). Furthermore, to our knowledge, no study has directly compared the strength of inter-individual Ne/ERN/PEA or accuracy relationships to intra-individual Ne/ERN/PEA relationships on the same sample size. For this reason, we strongly recommend that researchers report intra-individual relationships between the Ne/ERN and PEA, as often as possible (Table 2). Regarding PES, recommendations are unfortunately less clear. A variety of techniques have emerged to calculate post-error behavior (e.g., Dutilh et al., 2012; Schroder et al., 2020), with large variation in what researchers choose to report. There is still much debate about the best method is best to use (Pfister and Foerster, 2022). Therefore, when reporting PES, researchers should also consider reporting internal consistency, much like we recommend for the Ne/ERN. We also recommend that researchers continue to rigorously examine reliability measurements for each PES measure, as well as intra-individual analyses of brain-PES relationships as often as possible.

Finally, we here note that there may be other, subtler, ways to meaningfully analyze Ne/ERN-related behavior. For example, to our knowledge, no existing study has used drift diffusion models (e.g., Ratcliff, 1978) to understand behavioral outputs from the Ne/ERN. These models have been useful in understanding behavioral aspects of other ERP components. For example, previous work has found that the P3b amplitude represents some aspect of evidence accumulation—building to a certain threshold before a participant makes their response (Twomey et al., 2015). Therefore, it may be useful to examine whether drift-diffusion models of Ne/ERN amplitude might similarly relate to behavior. This may be especially relevant, considering that variables such as task emphasis (speed vs. accuracy) and OCD diagnosis can affect both Ne/ERN amplitude and drift-diffusion variables such as drift rate and boundary separation (Riesel et al., 2019). Furthermore, as briefly discussed earlier, researchers might also consider continuing to examine other, less “overt” behavioral modifications such as PERI. As we discuss in the next section, it is likely that some aspects of behavior modification exist beyond just the timing or accuracy of the discrete response button press itself.

### 5.3. Summary: How to choose which metrics to report and analyses to conduct

We have now discussed a variety of ways of reporting Ne/ERN-behavior relationships—from choices in level of analysis, to choices about which behavioral metric to report. While we strongly encourage work that specifically compares and examines all of these measurements together, we recognize that many Ne/ERN researchers may only be interested in addressing a partial aspect of this research question as an exploratory supplementary analysis to their main research question. Given the overwhelming number of options, how can researchers ensure that their analytical choices most closely answer their research question about Ne/ERN-behavior relationships?

First, a researcher must decide if what level of analysis they are interested in. For example, a researcher may wish to know whether individuals with anxiety have larger average Ne/ERN amplitudes. In this case, inter-individual analyses would be appropriate, though researchers should consider the internal consistency of participant and group-level ERPs (e.g., Clayson et al., 2020, 2021b). However, if a researcher wishes to understand whether a larger Ne/ERN on a given trial affects behavior on the next trial, intra-individual analyses may be a more appropriate match (e.g., Lim et al., 2009). To this end, centering techniques are a useful tool. Specifically, when examining how each Ne/ERN trial’s amplitude influences reaction time on the next trial, researchers should subtract each subject’s average Ne/ERN amplitude from each of the Ne/ERN amplitude trials (subject-mean centering). This allows one to separate out between-subjects, individual difference variation from the within-person, trial-to-trial variance (Enders and Tofighi, 2007), avoiding the “uninterpretable blend” (Raudenbush and Bryk, 2002, p. 139) of within- and between-subject contributions to the estimated fixed effect.

Second, a researcher must decide which behavior they are most interested in addressing. At the inter-individual level, researchers can examine both overall average accuracy and reaction time, as well as subject-level average PEA, PES, or PERI. At the intra-individual level, researchers can examine how Ne/ERN amplitude on a given trial affects accuracy on the next trial (single-trial PEA) or reaction time on the next trial (single-trial PES). Table 3 displays example research questions and subsequent analyses one could perform to answer them.

**TABLE 3:** How to choose Ne/ERN-behavior analyses based on research question.

Example research question	Recommended analysis
Does an individual’s average/“trait-level” Ne/ERN amplitude affect their overall accuracy or speed in responding?	Interindividual: examine how average Ne/ERN amplitude affects average reaction time and average accuracy/error rates.
Does an individual’s average/“trait-level” Ne/ERN amplitude affect their overall post-error behavior adjustment?	Interindividual: examine how average Ne/ERN amplitude affects average PES and average PEA
Does Ne/ERN amplitude on a given trial affect response time on the next trial?	Intra-individual: examine how single trial Ne/ERN amplitude affects reaction time on the next trial. With multiple subjects, researchers must use linear mixed-effects models and subject-mean center the data.
Does Ne/ERN amplitude on a given trial affect accuracy on the next trial?	Intra-individual: examine how single trial Ne/ERN amplitude affects accuracy on the next trial. Like the above analysis, researchers must use linear-mixed effects models and subject-mean center the data. Researchers answering this question must also use logistic regression.

### 5.4. Consideration of errors that are not two-choice button presses

Finally, another important decision that researchers must make is what kind of “errors” we include in our studies. Previously, some have argued that the Ne/ERN is difficult to link to behavior, because “the brain did not evolve to press E-Prime buttons” (e.g., Gehring and Knight, 2000; Gehring et al., 2018). Even in the event of pressing the wrong key button, the Ne/ERN occurs, by definition, at the onset of a motor error. Currently, the vast majority of Ne/ERN research occurs in the context of simple, two-choice response tasks, rather than in the context of more naturalistic movements seen in the real world. This approach to Ne/ERN research makes sense, given constraints on both EEG interpretability (i.e., being able to successfully separate out signal from muscle noise) and experimental feasibility. However, it is worth discussing the possibility that, in the context of goal-directed movement, the Ne/ERN reflects an early need to reconcile conflicting information from diverse input of both external sensory and internal efferent signals preceding motor emission at the output level (Pezzetta et al., 2018; Wessel, 2018).

Some work suggests the Ne/ERN is one aspect of a complex process aimed to remediate action, *via* detection and suppression of other motor function in certain contexts (Scheffers et al., 1996). Some behavioral studies of motor errors using pointing and reaching tasks have demonstrated that people are able to rapidly adapt their motor control and perceptual awareness to update internal predictions of sensory action outcomes, as the context of their choice changes with visual feedback of motor error (Synofzik et al., 2006). In theories of motor adaptation, downstream sensory-error processes help tailor the motor system to respond more appropriately on-line when the motor output differs from intended or predicted consequences of a motor command. In the presence of unexpected or erroneous outcome, it is thought that sensory feedback is required to update internal representations governing sensory processes (i.e., proprioception, perceptual awareness in limbs, Blakemore et al., 1999; Palidis et al., 2018). This and other evidence led some to argue that some of the fundamental designs of experimental factors have prevented an observation of continuous behavioral adaptation in response to errors (Payne et al., 2019).

Furthermore, electromyography (EMG) recorded even during a choice reaction-time task has also found some degree of sub-threshold corrective process. Using a color Simon task, Beatty et al. (2021) found that even when an overt correction was not made in response to an error, participants exhibited subthreshold corrective activity which interestingly, predicted post-error performance (i.e., compensatory behavior such as speeding or slowing) on subsequent trials. Similarly, as already discussed, some studies have found evidence of “partial” errors (e.g., Burle et al., 2008; Allain et al., 2009; Cohen and van Gaal, 2014; Ficarella et al., 2019), with some also finding a relationship between these partial errors and post-error behavior (Allain et al., 2009; Ficarella et al., 2019). These partial errors may explain the phenomenon of the correct-related negativity (CRN; Vidal et al., 2000, 2003) on high-conflict trials. These trials typically yield higher error rates, and it is logical to suspect that they may elicit more subthreshold errors as well. Finally, there is evidence of covert shifts in attentional processes before the advent of any discrete behavior (Steinhauser and Andersen, 2019), and increased activity in affective areas of the brain directly following errors (Marinkovic and Rosen, 2022), further demonstrating error-correction as a continuous and multi-step process.

These findings suggest that the Ne/ERN, and error-detection in general, is highly linked to goal-directed continuous movement. This is evolutionarily plausible, as ongoing sequences of motor events would be necessary to ensure survival (i.e., escaping a predator, picking berries from a bush). Inability to monitor for motor errors could, in this context, prove to be highly consequential. Therefore, studying the Ne/ERN in contexts related to more naturalistic continuous movements (rather than discrete, two-choice response tasks) may represent an opportunity for better understanding how the Ne/ERN relates to corrective behavior.

Fortunately, researchers have identified another frontal-central negative stability error ERP which is thought to be functionally and morphologically similar to the Ne/ERN, called balance-evoked N1 (Marlin et al., 2014). This balance-evoked N1 occurs in response to perturbations to balance, and researchers have argued its’ similarity in function to the Ne/ERN is likely due to identical neural substrates (Payne et al., 2019). Relative to the Ne/ERN, however, which is typically elicited in simple two-choice response tasks, the balance-evoked N1 has been linked to executive functioning for balance compensatory control across a variety of postures (i.e., sitting, standing, and walking) (Adkin et al., 2006; Marlin et al., 2014). Like the Ne/ERN’s ties to motivation, the amplitude of the balance N1 is contingent on the cognitive valuation of perceived consequence of balance control. Payne et al. (2019) explored similarities between the balance-evoked N1 as an adaptive motor error signal and the Ne/ERN to identify whether these function together as part of a common action monitoring system for behavioral adaptation. They highlighted parallels between the N1 and Ne/ERN, and emphasized the potential benefit of leveraging overlap in these components to more precisely control the type and sequencing of errors in future experimental designs. Therefore, the N1 represents a useful option for researchers interested in studying error-behavior relationships in more naturalistic settings.

In summary, there is compelling evidence to suggest that error-processing and detection are processes that may have evolved to detect deviations in continuous movements, rather than just strictly differences in discretely correct or incorrect movements (e.g., Payne et al., 2019). These findings could explain some of the discrepancies in the CRN literature reviewed above—it is possible that the CRN does not index conflict *per se*, but rather partial errors. It is possible that certain paradigms would elicit more of these such partial errors, and this is more likely to occur on higher conflict, difficult trials. All of these insights are missed when only considering errors as a binary outcome. Therefore, in addition to traditional Ne/ERN paradigms such as the flanker task and Stroop task, errors should be examined in paradigms that allow for more naturalistic movement. At the very least, future work should continue to use EMG to study how brain activity relates to the initiation of incorrect movement. Indeed, these instances should be of high interest to those who wish to understand how the brain corrects for mistakes after recognizing them—as the brain is recognizing and correcting the error in real time.

## 6. Conclusion and future directions

**FIGURE 1:**
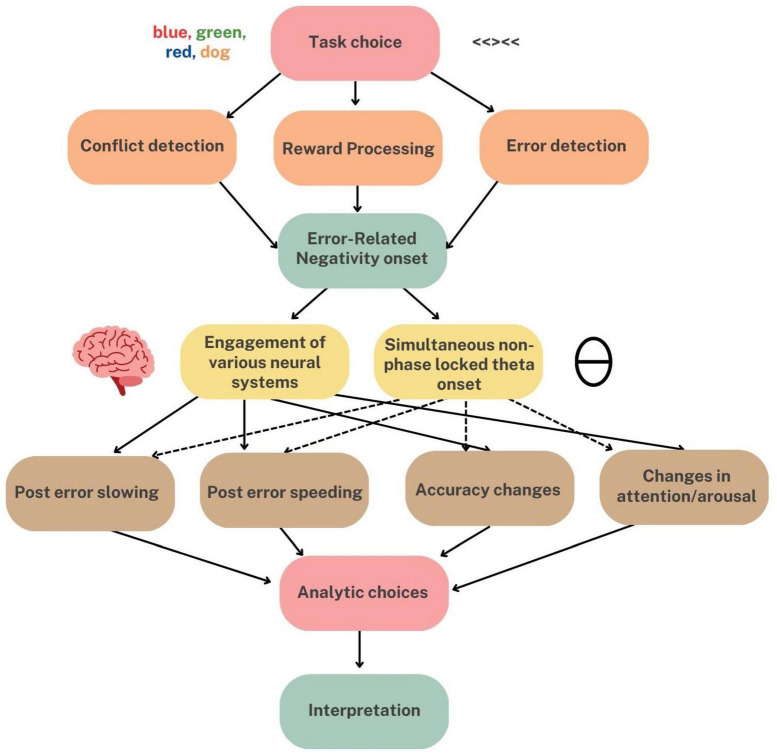
Example of potential pathways that can influence our interpretations of the relationship between Ne/ERN and behavior. For example, one task might engage underlying “conflict detection” systems more so than error detection to produce an Ne/ERN. From here, differential neural systems might be recruited to implement a variety of behavioral adjustments. Lastly, researcher analytical choices (i.e., pre-processing, number of errors included) can still influence interpretations. This graphic is intended to depict the numerous pathways that can exist to influence results, even within one task paradigm. Original graphic created using Canva.com (© *via* Canva.com).

The task of relating characteristics of the Ne/ERN to behavioral outcomes has challenged and engaged cognitive electrophysiologists for decades. In the present manuscript, we argue that delineating a relationship between the Ne/ERN and behavioral adjustment is challenging because error-processing is a multiply implicated process, with many inputs, outputs, and moderators. We summarize these multiple factors in an example illustration in Figure 1. In summary, we argue that the link between the Ne/ERN and behavior is likely difficult to parse in part because of experimental factors such as task selection and analytic choices, and in part because of neurobiological factors, such as the Ne/ERN’s involvement in larger, more generalized cognitive control networks. It is worth noting that these experimental factors likely exacerbate discrepancies caused by the Ne/ERN’s widespread distribution in the brain. For example, it is possible that an Ne/ERN elicited by one choice of task may tap into a different control network than an Ne/ERN elicited by another choice in task. All of this points to one overarching argument—the behavioral utility of the Ne/ERN is likely largely contextual. As error-processing is a very generalized process, we must recognize that it is likely a computationally complex problem, with multiple input and output conditions.

Yet, despite these challenges, understanding how error-processing relates to behavior is still an important endeavor to undertake. Answering this research question will allow us to further understand the extent to which increased levels of error-processing is adaptive or maladaptive. While there is little we can do about the inherent neural structures underlying error-processing, there is much that researchers can do to control the experimental factors that make the Ne/ERN’s relationship to behavior such a thorny issue to tackle. In Table 2, we summarize these challenges and considerations, and our recommended solutions. We encourage researchers to consider using this table as somewhat of a checklist when reporting Ne/ERN results—particularly for studies that report the Ne/ERN’s relationship to behavior. We encourage all researchers to use this checklist as a resource, and to report Ne/ERN-behavior relationships whenever possible, even if their research question is not explicitly about behavior. However, for those researchers who are explicitly interested in the Ne/ERN’s relationships to behavior adjustment, as we have covered in the manuscript, there are many existing questions to address. In Box 1, we summarize what are, in our opinion, some of the more pressing questions about Ne/ERN and behavior.

As we have outlined in the present manuscript, error-processing is a complex, and multiply determined process in the brain. As such, we will have to work to ensure that we acknowledge this as we design studies and make analytical choices when examining the Ne/ERN. Doing so will enable us to better understand whether the Ne/ERN truly does implement corrective behavior, if it is simply an affective and aversive response to errors, or both.

## Author contributions

All authors listed have made a substantial, direct, and intellectual contribution to the work, and approved it for publication.

## Footnotes

1 While it is beyond the scope of this manuscript to extensively review further evidence for these two dissociated networks, and how they relate to other similar conceptualizations of attention networks readers can refer to Gratton et al. (2018).
